# Integrative analysis of the metabolome and transcriptome provides insights into the mechanisms of lignan biosynthesis in *Herpetospermum pedunculosum* (Cucurbitaceae)

**DOI:** 10.1186/s12864-024-10306-1

**Published:** 2024-04-29

**Authors:** Ziwei Zhu, Daihan Chen, Min Sun, Maotao Xiao, Peng Huang, Dongsheng Ren, Yixi Yang, Zhen Zhang, Qi Zhao, Rui Li

**Affiliations:** 1 Engineering Research Center of Sichuan-Tibet Traditional Medicinal Plant, Chengdu University, 610106 Chengdu, China; 2 Institute for Advanced Study, Chengdu University, 610106 Chengdu, China; 3 Pharmacy College, Chengdu University of Traditional Chinese Medicine, 611137 Chengdu, China; 4 School of Food and Biological Engineering, Chengdu University, 610106 Chengdu, China; 5 Tibet Rhodiola Pharmaceutical Holding Company, 850000 Lhasa, China

**Keywords:** *Herpetospermum Pedunculosum*, Seed developmental stages, Lignan biosynthesis, Metabolome, Transcriptome

## Abstract

**Background:**

*Herpetospermum pedunculosum * (Ser.) C. B. Clarke is a traditional Chinese herbal medicine that heavily relies on the lignans found in its dried ripe seeds (*Herpetospermum caudigerum*), which have antioxidant and hepatoprotective functions. However, little is known regarding the lignan biosynthesis in *H. pedunculosum*. In this study, we used metabolomic (non-targeted UHPLC-MS/MS) and transcriptome (RNA-Seq) analyses to identify key metabolites and genes (both structural and regulatory) associated with lignan production during the green mature (GM) and yellow mature (YM) stages of *H. pedunculosum*.

**Results:**

The contents of 26 lignan-related metabolites and the expression of 30 genes involved in the lignan pathway differed considerably between the GM and YM stages; most of them were more highly expressed in YM than in GM. UPLC-Q-TOF/MS confirmed that three *Herpetospermum*-specific lignans (including herpetrione, herpetotriol, and herpetin) were found in YM, but were not detected in GM. In addition, we proposed a lignan biosynthesis pathway for *H. pedunculosum* based on the fundamental principles of chemistry and biosynthesis. An integrated study of the transcriptome and metabolome identified several transcription factors, including HpGAF1, HpHSFB3, and HpWOX1, that were highly correlated with the metabolism of lignan compounds during seed ripening. Furthermore, functional validation assays revealed that the enzyme 4-Coumarate: CoA ligase (4CL) catalyzes the synthesis of hydroxycinnamate CoA esters.

**Conclusion:**

These results will deepen our understanding of seed lignan biosynthesis and establish a theoretical basis for molecular breeding of *H. pedunculosum*.

**Supplementary Information:**

The online version contains supplementary material available at 10.1186/s12864-024-10306-1.

## Background

*Herpetospermum pedunculosum* (Ser.) C. B. Clarke, belonging to the family Cucurbitaceae, is a traditional Tibet medicinal herb. The dried ripe seeds of *H. pedunculosum* (*Herpetospermum caudigerum* Wall), with “Se-ji-mei-duo” in Tibet or Chinese name “Bo-Leng-Gua-Zi,” are frequently used for the treatment of “Chiba” diseases caused by liver dysfunction, such as jaundice, hepatitis, cholecystitis [1]. Modern pharmacological studies have indicated that *H. caudigerum* contains a variety of bioactive lignans, such as herpetin, herpetfluorenone, and herpetone [2, 3], which are mainly responsible for their anti-infammatory [1], anti-fatigue [4], anti-hepatitis B virus [5], anti-cholestatic [6], and hepatoprotective activities [7–9] ascribed to them. The use of *H. pedunculosum* in medicine has led to an increasing trend of using this plant as the main ingredient in various medications, such as the “Shi-wei-di-da” capsule, “twenty-five flavors-big soup” pills, and “Jiu-wei-Niu-huang” pills [9]. However, the active ingredients of medicinal plants are generally present at a lower content. Recent studies on *H. pedunculosum* have primarily focused on exploring its active ingredients and pharmacological mechanisms [9–11]. However, the mechanisms involved in lignan biosynthesis and regulation in *H. pedunculosum* have not yet been elucidated.

Lignans are polyphenolic compounds that are widespread in plants and often play a role in biological defense and growth [12]. All lignans are derived from the phenylpropanoid monomeric unit precursor coniferyl alcohol, which can be produced through the general phenylpropanoid pathway [13]. Lignan is produced through the initial dimerization of the lignan backbone coniferyl alcohol into various intermediates, followed by post-dimerization transformations involving methylation, oxidation, demethylation, cyclization, hydrolysis, and/or hydroxylation [14]. The content and composition of lignans are significantly influenced by genetic and environmental factors as well as other variables, including the maturity stage of the seeds and fruits [15]. In *Schisandra chinensis*, long-read transcriptome sequencing analysis revealed significant variations in lignan biosynthesis during fruit development between the green- and red-colored berry stages, which indicates that ripened fruits contain high levels of lignans [16]. Throughout the maturation process of flax seeds, lignan concentration consistently increases from the initial stage to the mature stage [17]. The number of lignans in *H. pedunculosum* seeds is also known to be influenced by seed maturity [18].

In recent years, transcriptomics and metabolomics have been intensively utilized to screen lignan biosynthesis-related metabolites and genes [19–21]. Although several structural genes and transcription factors implicated in lignan biosynthesis and regulation have been identified in many plants, the regulation of species-specific lignan biosynthesis pathways has not yet been fully elucidated. In this study, integrative analysis of the metabolome and transcriptome was used to examine differences in *H. pedunculosum* lignans and the expression patterns of key genes involved in lignan biosynthesis between the green mature stage (GM) and yellow mature stage (YM) during maturation. These findings will facilitate a better understanding of the synthesis mechanism of lignans in seeds and provide insights into the metabolic engineering of lignans in *H. pedunculosum*.

## Results

### Metabolomic profiling and UPLC-Q-TOF/MS analyses

**Fig. 1:**
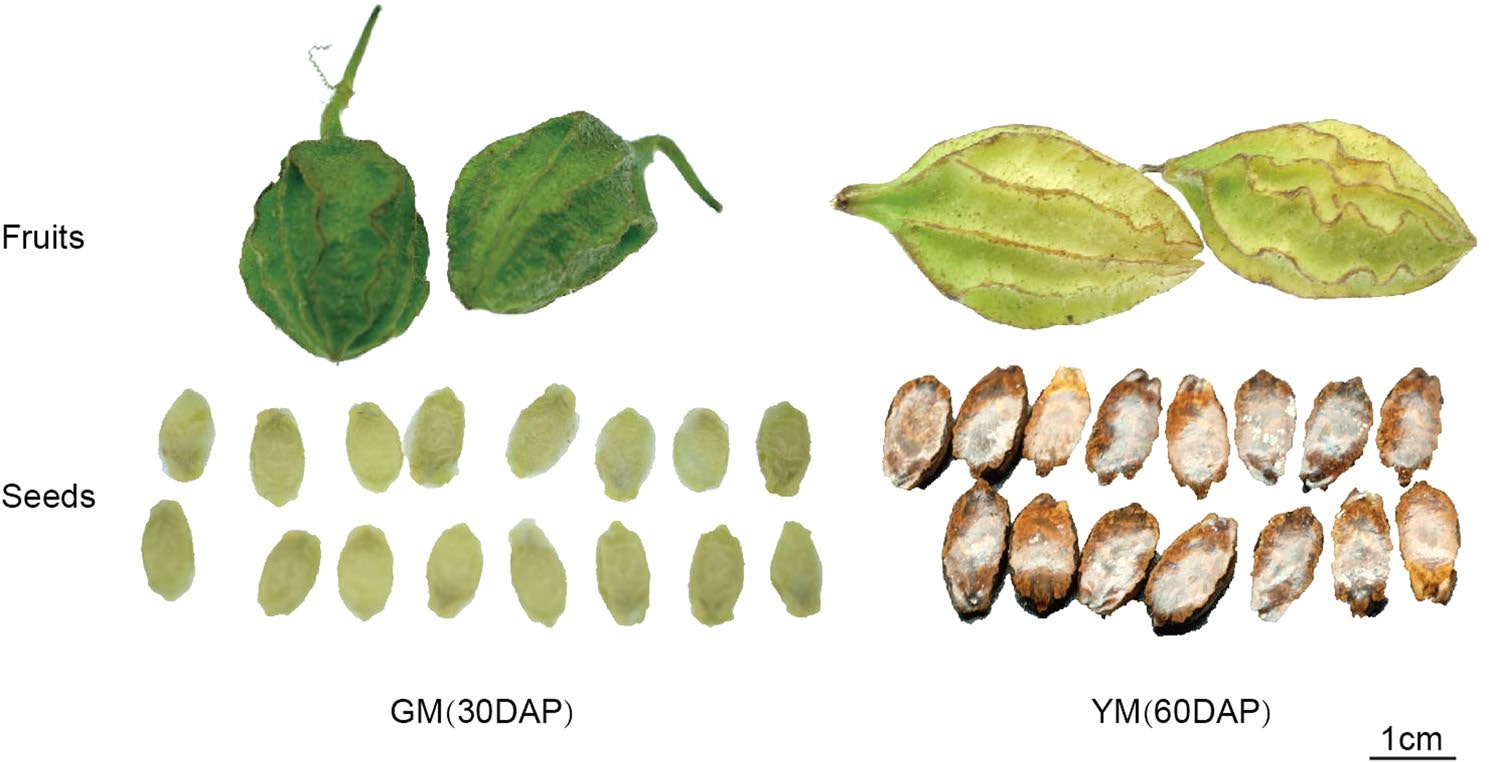
Morphological observations of *H. pedunculosum* fruit and seed developmental stages. Seeds of *H. pedunculosum* were green at the green mature stage (30 days after pollination, 30 DAP) and black at the yellow mature stage (60 days after pollination, 60 DAP)

**Fig. 2:**
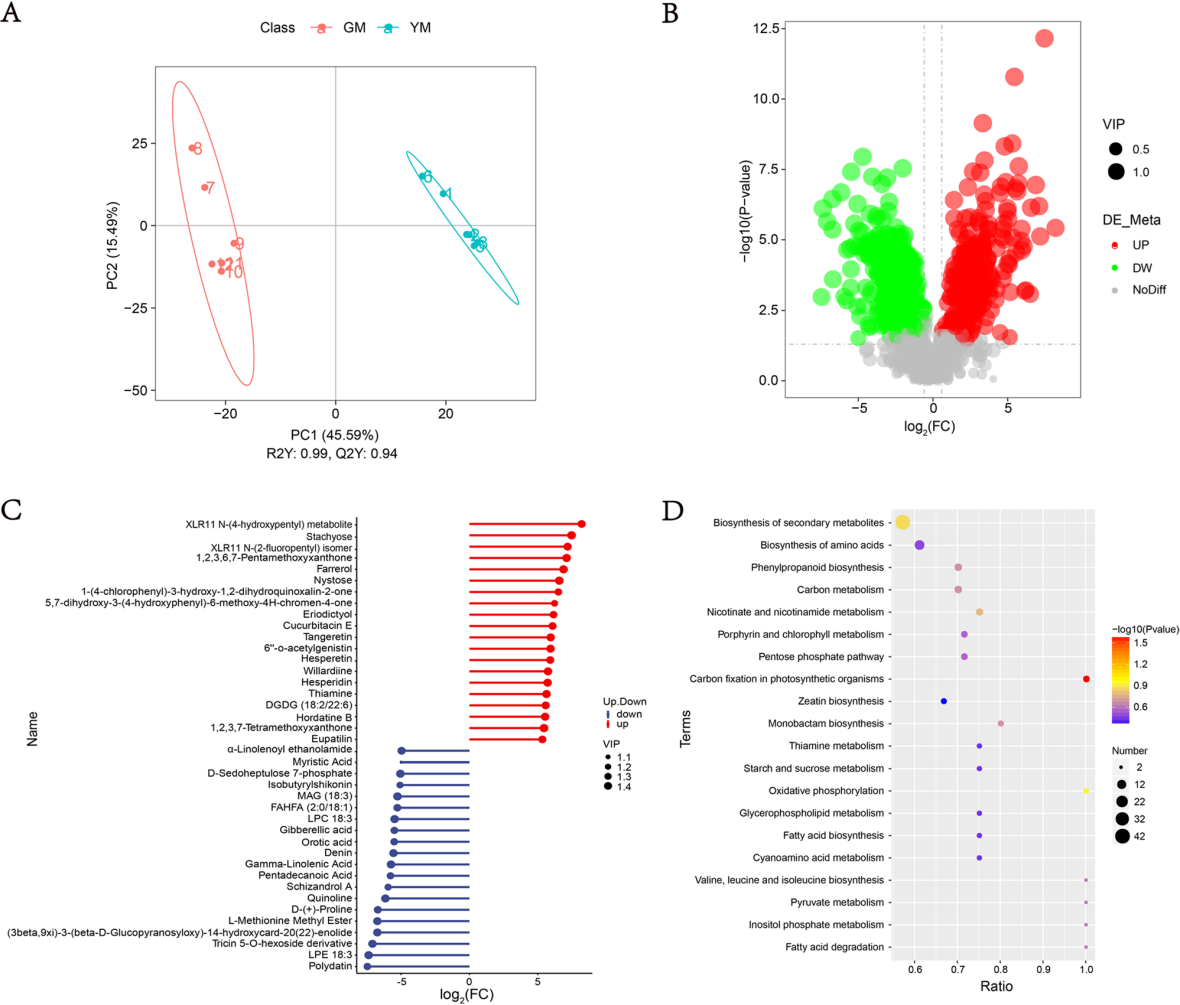
Preliminary analysis of metabolomic data for YM vs. GM. **A**. PLS-DA score plot; **B**. Volcano plot of DAMs; **C**. Stem plot of the top 40 DAMs; **D**. Top 20 KEGG enriched pathways

The seeds of *H. pedunculosum* underwent two different stages of maturation according to melon color and seed morphology (Fig. 1). To investigate the metabolite dynamics during this process, we used an untargeted metabolic comparison at two stages (GM to YM). Partial least squares-discriminant analysis (PLS-DA) score plots revealed clear differences between GM and YM groups (Fig. 2A). Using VIP > 1 and|log_2_(Fold Change)| > 1 as screening criteria for differentially accumulated metabolites (DAMs), we identified 565 DAMs (252 upregulated and 313 downregulated) in YM vs. GM (Fig. 2B). The profiles of the top 40 DAMs between the two seed development stages are shown in Fig. 2C and Dataset S1. The metabolites in GM and YM exhibited distinct accumulation trends. Compared with GM, YM showed a high accumulation of phenylpropanoid compounds, whereas the level of lipid compounds decreased as the seeds ripened. KEGG enrichment analysis indicated that the DAMs were mainly enriched in pathways, including the phenylpropanoid metabolic pathway and biosynthesis of secondary metabolites (Fig. 2D). To further investigate lignan biosynthesis during seed development, twenty-six DAMs involved in the lignan pathway were identified according to KEGG (phenylpropanoid biosynthesis, ko00940) and the literature. Among them, 19 metabolites were upregulated and seven were downregulated in YM (Table 1). *p*-coumaric acid, sinapinic acid, and coniferaldehyde (ferulaldehyde) exhibited considerably higher accumulations in YM, indicating that these compounds play important roles in lignan biosynthesis in *H. pedunculosum*. Additionally, eight lignan metabolites with significant differential expressions were identified. Six of these were upregulated in YM, including eleutheroside E, schisantherin E, syringin (eleutheroside B), schisantherin A, secoisolariciresinol, and schizandrol B.

**Fig. 3:**
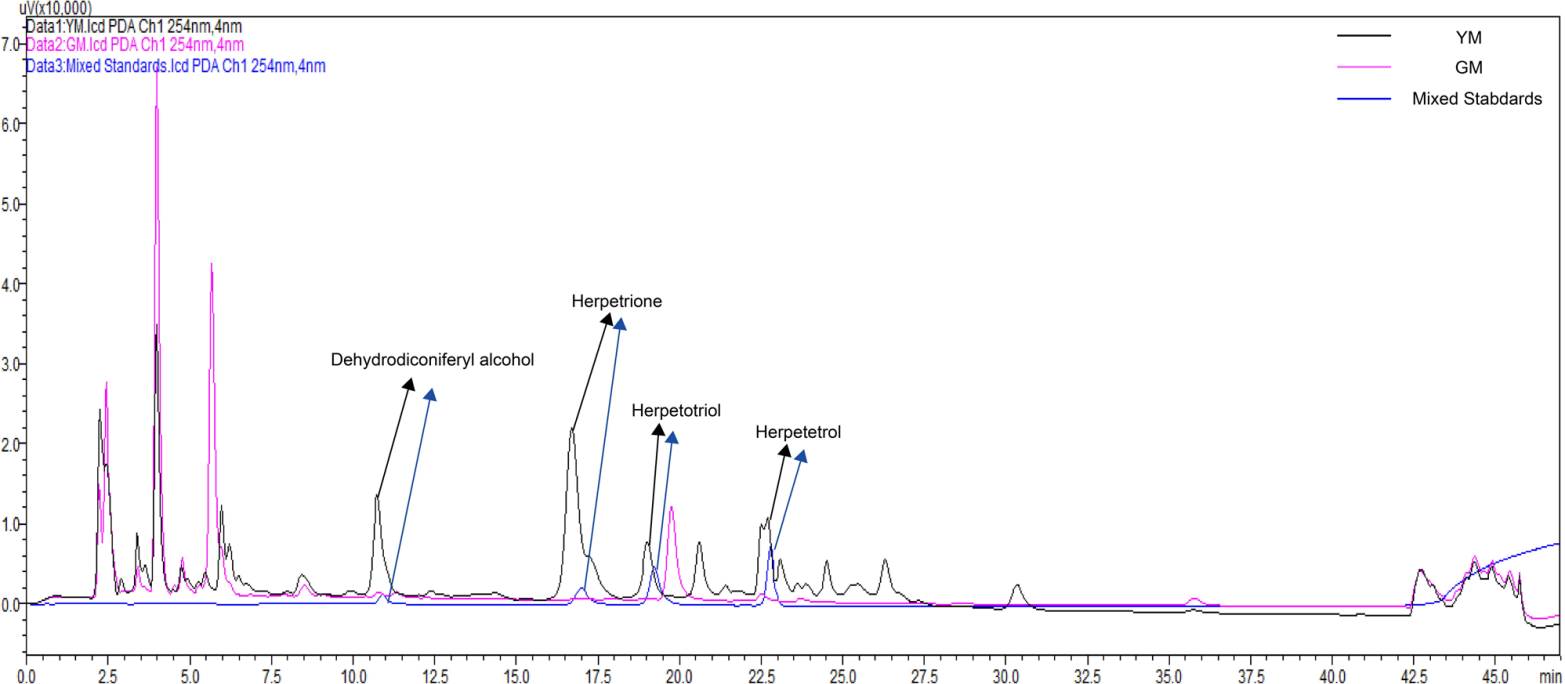
UPLC-Q-TOF/MS chromatogram of lignans extracted from GM and YM of *H. pedunculosum*

Given that the untargeted metabolome could not identify *Herpetospermum*-specific lignans, we used UPLC-Q-TOF/MS to detect lignans from the two seed developmental phases, with four standard lignans serving as standards for quantification. As shown in Fig. 3, all identified *Herpetospermum*-specific lignans were high in YM but not in GM. Herpetrione was the most commonly detected lignan in the YM. These results indicate that *Herpetospermum*-specific lignans mainly accumulated during the seed maturation stage.

**Table 1:** List of lignan biosynthesis-related metabolites in YM. vs. GM

Compound name	Formula	Retention time (min)	VIP value	Log_2_ (FC)	Type
Eleutheroside E	C_34_H_46_O_18_	5.40	1.47	4.98	up
Schisantherin E	C_30_H_34_O_9_	5.67	1.37	3.77	up
Syringin (Eleutheroside B)	C_17_H_24_O_9_	5.44	1.30	3.35	up
Columbianadin	C_19_H_20_O_5_	4.87	1.40	3.28	up
Sinapinic acid	C_11_H_12_O_5_	5.26	1.38	3.27	up
Schisantherin A	C_30_H_32_O_9_	5.73	1.31	3.08	up
Sauchinone	C_20_H_20_O_6_	5.86	1.34	2.86	up
Coniferaldehyde (Ferulaldehyde)	C_10_H_10_O_3_	5.47	1.45	2.34	up
Isoferulic Acid	C_10_H_10_O_4_	5.57	1.25	2.18	up
*O*-*p*-Coumaroyl quinacyl quinic acid *O*-hexoside	C_29_H_38_O_18_	5.62	1.27	2.09	up
Secoisolariciresinol	C_20_H_26_O_6_	5.61	1.27	2.04	up
3-*O*-*p*-coumaroyl shikimic acid *O*-hexoside	C_22_H_26_O_12_	1.37	1.39	1.83	up
Schizandrol B	C_23_H_28_O_7_	6.14	1.15	1.65	up
Trans-Anethole	C_10_H_12_O	5.41	1.15	1.56	up
4-*p*-Coumaroylquinic acid	C_16_H_18_O_8_	5.30	1.12	1.52	up
3-Methoxycinnamic acid	C_10_H_10_O_3_	5.63	1.24	1.43	up
Angeloyl-(+)-gomisin K3	C_28_H_36_O_7_	8.37	1.07	1.41	up
*p*-Coumaric acid	C_9_H_8_O_3_	5.56	1.09	1.33	up
4-Methoxycinnamic acid	C_10_H_10_O_3_	5.22	1.15	−1.42	down
*L*-Phenylalanine	C_9_H_11_NO_2_	4.92	1.35	−1.49	down
2-Hydroxycinnamic acid	C_9_H_8_O_3_	2.45	1.34	−1.60	down
Nordihydroguaiaretic acid	C_18_H_22_O_4_	5.88	1.43	−2.20	down
Coniferin	C_16_H_22_O_8_	5.20	1.17	−2.31	down
Gomisin D	C_28_H_34_O_10_	6.29	1.35	−2.40	down
Magnolol	C_18_H_18_O_2_	5.70	1.36	−2.98	down

### Transcriptomic profiling

**Fig. 4:**
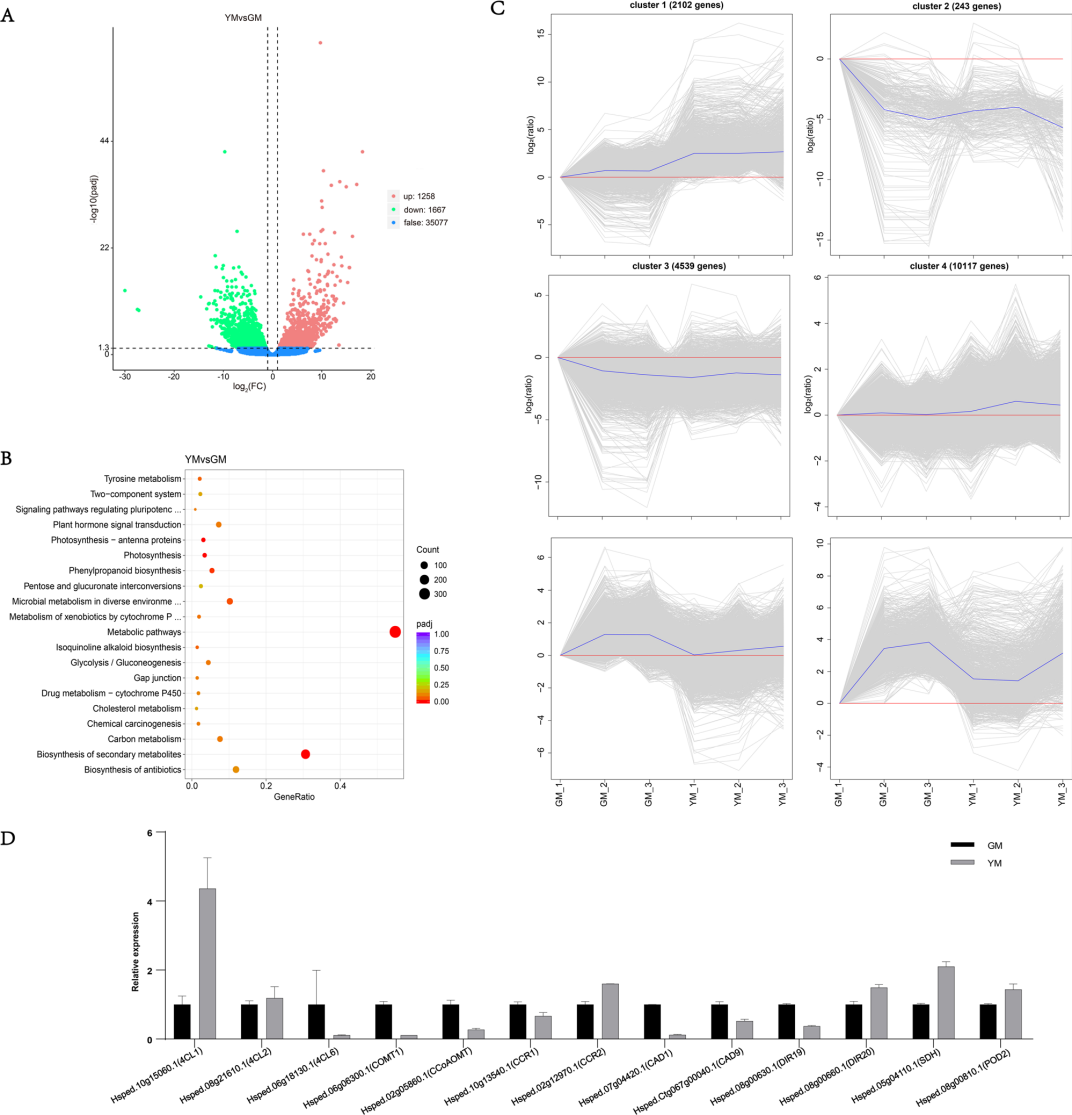
RNA-Seq and qRT-PCR analyses of DEGs in YM vs. GM. **A**. Volcano plot of DEGs in YM vs. GM; **B**. Top 20 KEGG enriched pathways; **C**. Six subclusters of 27,000 DEGs were clustered. The blue line shows the average values of the relative expression levels in each sub-cluster, and the gray lines represent the relative expression levels of each gene in each sub-cluster. **D**. qRT-PCR analysis

To uncover the potential molecular basis of lignan-related compound variations in *H. pedunculosum* development, a transcriptomic comparison of the YM and GM stages was performed using RNA-seq technology. Six RNA libraries produced 38.17 Gb Clean data, > 6.2 Gb per library, with a percentage of Q30 bases exceeding 91% (Table S2). In total, 2925 DEGs were detected in YM vs. GM (1258 upregulated; 1667 downregulated) (Fig. 4A). According to KEGG analysis, the significant DEGs between the YM and GM libraries were mainly enriched in the “metabolic pathways”, “biosynthesis of secondary metabolites”, “Photosynthesis” and “Phenylpropanoid biosynthesis” (Fig. 4B, Dataset S2). The phenylpropanoid pathway is involved in the lignan biosynthesis in plants. The expression of 141 candidate unigenes was involved in the phenylpropanoid biosynthesis pathway (ko00940). Among them, 30 significant DEGs that controlled lignan biosynthesis were obtained from YM vs. GM, including EC: 2.1.1.68 (caffeic acid 3-*O*-methyltransferase, COMT), EC: 2.1.1.104 (caffeoyl-CoA *O*-methyltransferase, CCoAOMT), EC: 6.2.1.12 (4-coumarate–CoA ligase, 4CL), EC: 1.2.1.44 (cinnamoyl-CoA reductase, CCR), and EC: 2.3.1.133 (shikimate *O*-hydroxycinnamoyltransferase, HCT). H-means clustering analysis revealed six distinct clusters (Fig. 4C). The upregulated DEGs in cluster 1 were mainly enriched in the metabolic pathway, followed by the biosynthesis of secondary metabolites. To validate the transcriptome data, we performed qRT-PCR to analyze the expression of 12 randomly selected genes involved in the lignan biosynthesis pathway. The results indicated that the expression patterns of these 12 genes were consistent with their transcriptome expression profiles (Fig. 4D, Dataset S4).

### Integrated analysis of the metabolome and transcriptome

**Fig. 5:**
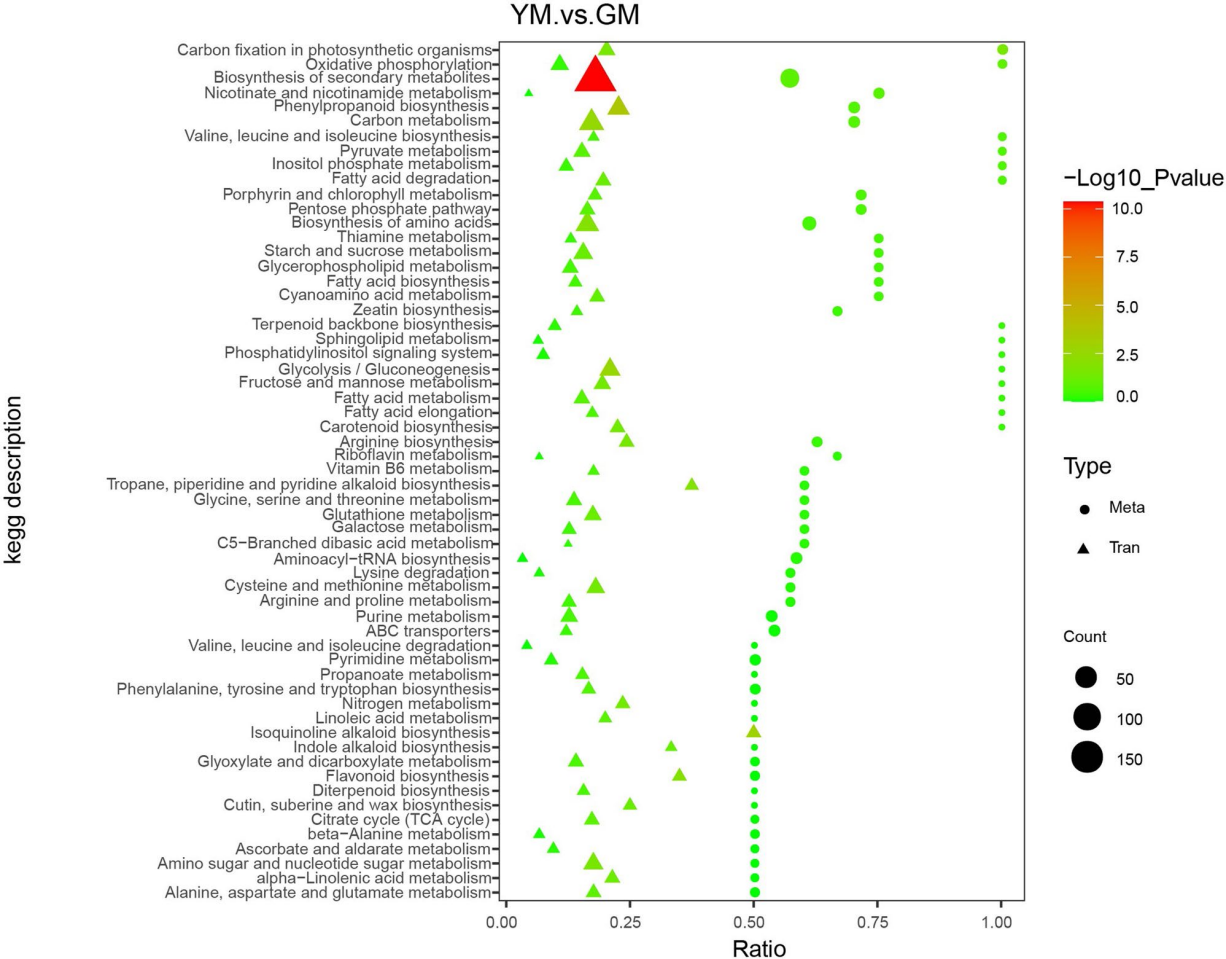
KEGG analysis of DAMs and DEGs enriched in the same pathway

**Fig. 6:**
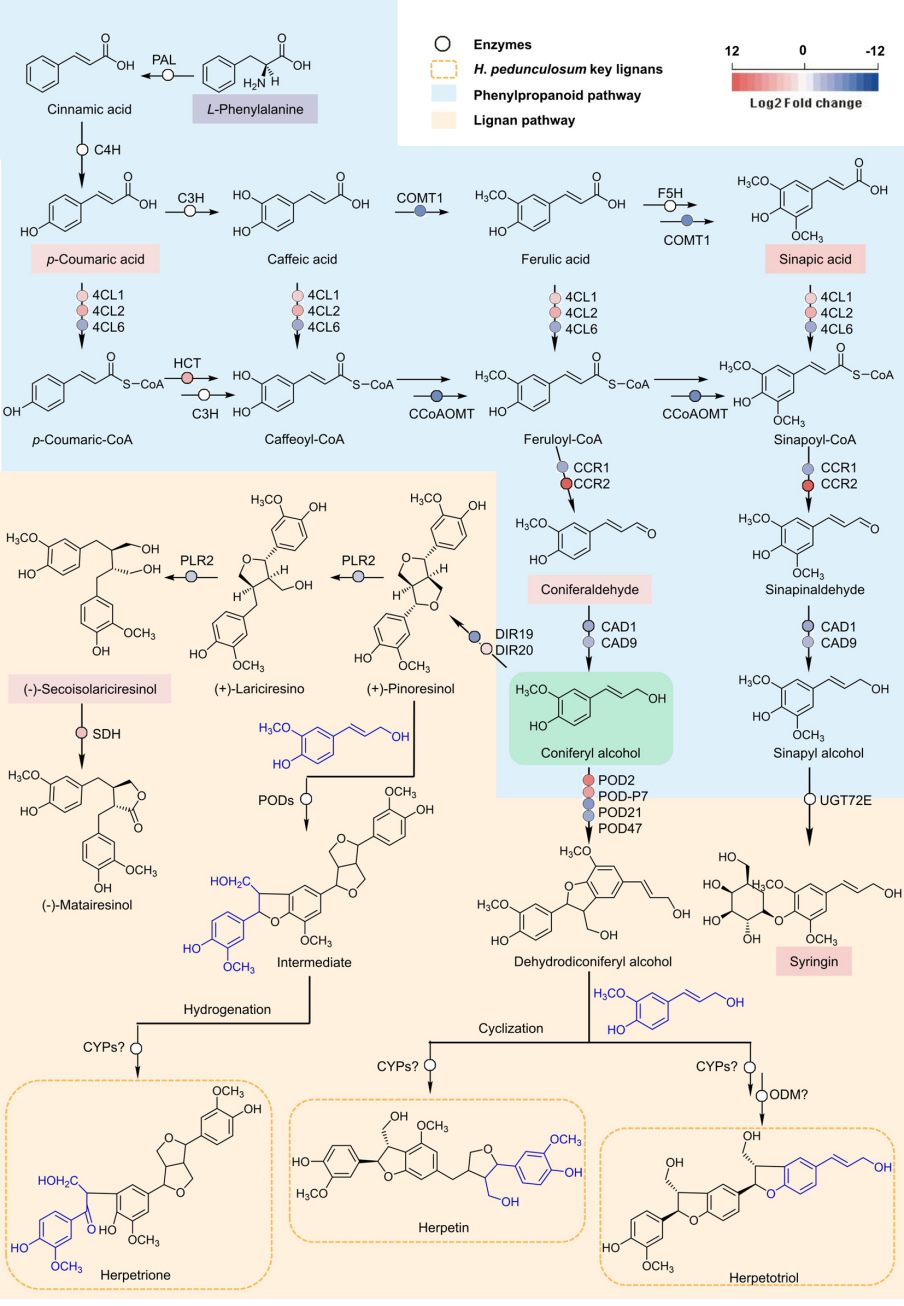
Schematic diagram of the putative pathway for lignan biosynthesis in *H. pedunculosum*. Rectangles and circles marked with blue and red backgrounds represent reduced and increased levels of DAMs and DEGs, respectively. PAL, phenylalanine ammonia-lyase; C4H, cinnamate 4-hydroxylase; C3H, cinnamate 3-hydroxylase; COMT, caffeic acid 3-omethyltransferase; F5H, ferulate-5-hydroxylase; 4CL, 4-coumarate: CoA ligase; HCT, *p*-hydroxycinnamoyl-CoA shikimate/quinate hydroxycinnamoyl transferase; CCoAoMT, caffeoyl-CoA *O*-methyltransferase; CCR, cinnamoyl-CoA reductase; CAD, cinnamyl alcohol dehydrogenase; DIR, dirgent; PLR, pinoresinol/lariciresinol reductase; SDH, secoisolariciresinol dehydrogenase; POD, peroxidase; CYP, cytochromeP450; ODM, *O*-demethylase; UGT72E, UDP-glycosyltransferase 72E

The KEGG database enrichment results for DEGs and DAMs showed that several DEGs and DAMs were enriched in the same KEGG pathway, including carbon fixation in photosynthetic organisms, oxidative phosphorylation, biosynthesis of secondary metabolites, nicotinate and nicotinamide metabolism, and phenylpropanoid biosynthesis (Fig. 5, Dataset S3). We constructed a rough metabolic pathway map related to *H. pedunculosum* lignan biosynthesis based on the KEGG database and the literature (Fig. 6). In general, the phenylpropanoid and lignan pathways combined to form lignan in *H. pedunculosum* seeds. The general phenylpropanoid pathway begins with *L*-phenylalanine, catalyzed by phenylalanine ammonia lyase (PAL) and cinnamate 4-hydroxylase (C4H), leading to the synthesis of *p*-coumaric acid, which offers a crucial branch point for the production of diverse metabolites, including lignans, lignins, and flavonoids [22]. *p*-coumaric acid serves as a substrate that undergoes a cascade of chemical conversions along with the formation of metabolite intermediates, such as *p*-coumaric CoA, caffeic acid, caffeoyl-CoA, ferulic acid, feruloyl-CoA, coniferaldehyde, and coniferyl alcohol. 4CL, cinnamate 3-hydroxylase (C3H), COMT, CCoAOMT, HCT, CCR, and cinnamyl alcohol dehydrogenase (CAD) are involved in this process. The majority of the genes encoding enzymes implicated in the general phenylpropanoid pathway (phenylalanine to coniferyl alcohol) were found in the transcriptome. Four DEGs, *4CL1* (Hsped.10g15060.1), *4CL2* (Hsped.08g21610.1), *HCT* (Hsped.10g00710.1), and *CCR2* (Hsped.02g12970.1), were upregulated, and six DEGs were downregulated in YM (Fig. 6, Dataset S4), which may be attributed to the intricate regulatory mechanisms governing secondary metabolites. Among these, 4CL is an important enzyme in phenylpropanoid biosynthesis and affects lignan biosynthesis. Three DEGs encoding 4CL were identified, two of which were upregulated and one was downregulated in YM, suggesting that multiple isoforms and paralogous genes may perform redundant functions.

In subsequent synthesis, coniferyl alcohol serves as an important starting material for the synthesis of *Herpetospermum*-specific lignans (including herpetrione, herpetotriol, and herpetin), which is a very complex synthesis process controlled by numerous genes and enzymes. Pinoresinol is an important intermediate in the formation of herpetrione, which is obtained through the oxidation and cyclization of two molecules of coniferyl alcohol in the presence of dirigent protein (DIR) [23]. Once Pinoresinol is formed, it undergoes cyclization and oxidation with another molecule of coniferyl alcohol to obtain another intermediate. The formed intermediate is finally hydrolyzed and oxidized to obtain herpetrione. Dehydrodiconiferyl alcohol is also an important intermediate in the formation of *Herpetospermum*-specific lignans and is produced directly from the dimerization of a soluble intracellular peroxidase [24]. After reacting with coniferyl alcohol, dehydrodiconiferyl alcohol may be transferred to herpetin after a series of rearrangement and oxidation reactions (methyl migration, demethoxylation, addition/oxidation, and methoxylation) and transferred to herpetotriol catalyzed by Cytochrome P450 (CYPs) and *O*-demethylase (ODM). However, the specific enzymes involved in this process remain to be identified. Here, we identified two DIR-encoding DEGs that control the rate-limiting stage of lignan biosynthesis and found that *DIR20* (Hsped.08g00660.1) was more highly expressed in YM than in GM. In addition, certain candidate unigenes related to the synthesis of matairesinol (*secoisolariciresinol dehydrogenase*, Hsped.05g04110.1) and etoposide (*CYP82D61*, Hsped.01g17190.1) were identified in this study [25], the expression of these DEGs was upregulated in YM compared with GM. Overall, the pathway demonstrated that metabolites such as *p*-coumaric CoA, coniferaldehyde, and secoisolariciresinol accumulated significantly in YM, possibly explaining the higher lignan accumulation in YM. These results revealed that these differentially expressed enzymes and metabolites may be involved in lignan biosynthesis in ripe seeds.

**Fig. 7:**
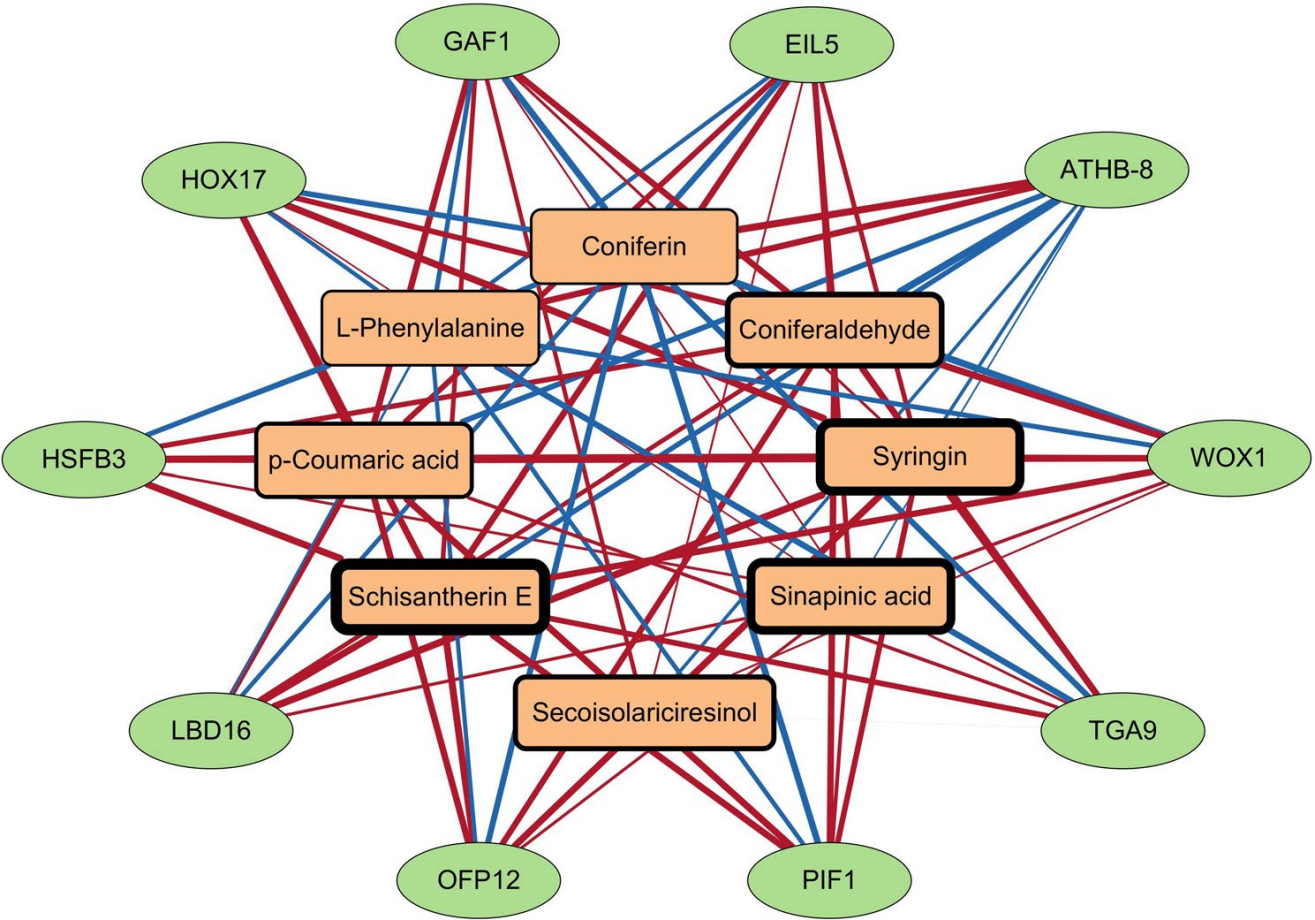
Connection network between DAMs (yellow rectangles) and TFs (Green circles). EIL5, Hsped.09g10850.1; ATHB-8, Hsped.01g22160.1; WOX1, Hsped.09g03880.1; TGA9, Hsped.07g12960.1; PIF1, Hsped.06g18730.1; OFP12, Hsped.03g02030.1; LBD16, Hsped.06g19360.1; HSFB3, Hsped.05g08010.1; HOX17, Hsped.09g04300.1; GAF1, Hsped.10g06590.1

Furthermore, the TF might also regulate lignan synthesis during *H. pedunculosum* seed development. Using correlation network analysis, ten TFs were found to be significantly associated with lignan-related metabolites (Dataset S5). Eight lignan-related metabolites and ten TFs generated 76 subnetworks (|Pearson correlation coefficient|>0.83, *P* < 0.01) (Fig. 7). The subnetwork revealed that several single lignan-related metabolites were regulated by multiple TFs or that single TFs regulated multiple lignan-related metabolites. For example, *L*-phenylalanine was found to be negatively associated with all ten TFs, except for *ATHB-8*. Secoisolariciresinol and schisantherin E were significantly positively associated with transcripts annotated as *GAF1*, *WOX1*, *EIL5*, *PIF1*, and *TGA9*. Ten TFs were significantly correlated with many metabolites but their regulatory relationships require further study.

### Functional validation of Hp4CL1 and Hp4CL2

**Fig. 8:**
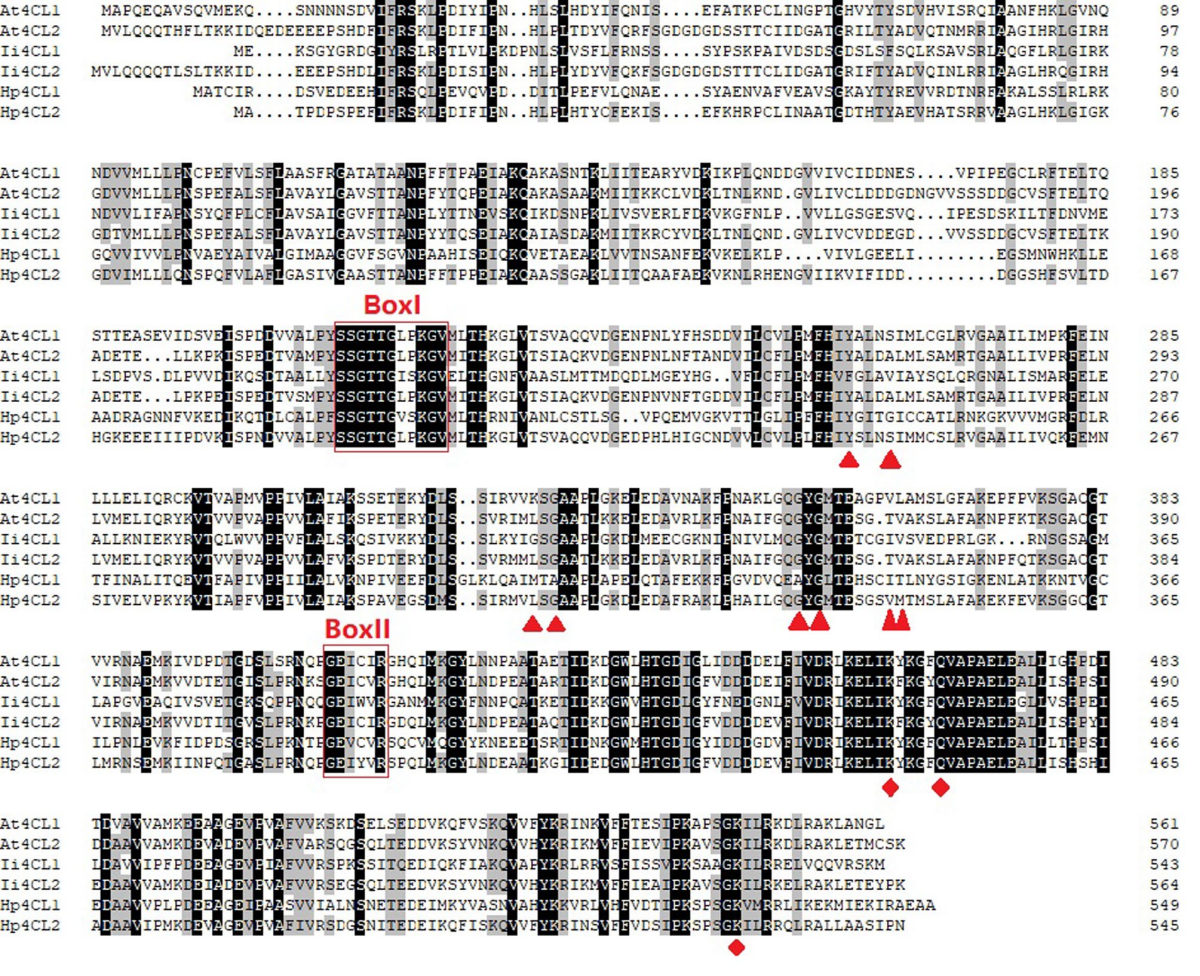
Amino acid sequence alignment of Hp4CL with other plant 4CLs. Box: conserved motifs; triangles: residues involved in hydroxycinnamate binding; diamonds: residues involved in enzymatic functions. At: *Arabidopsis thaliana*; Ii: *Isatis indigotica*

**Fig. 9:**
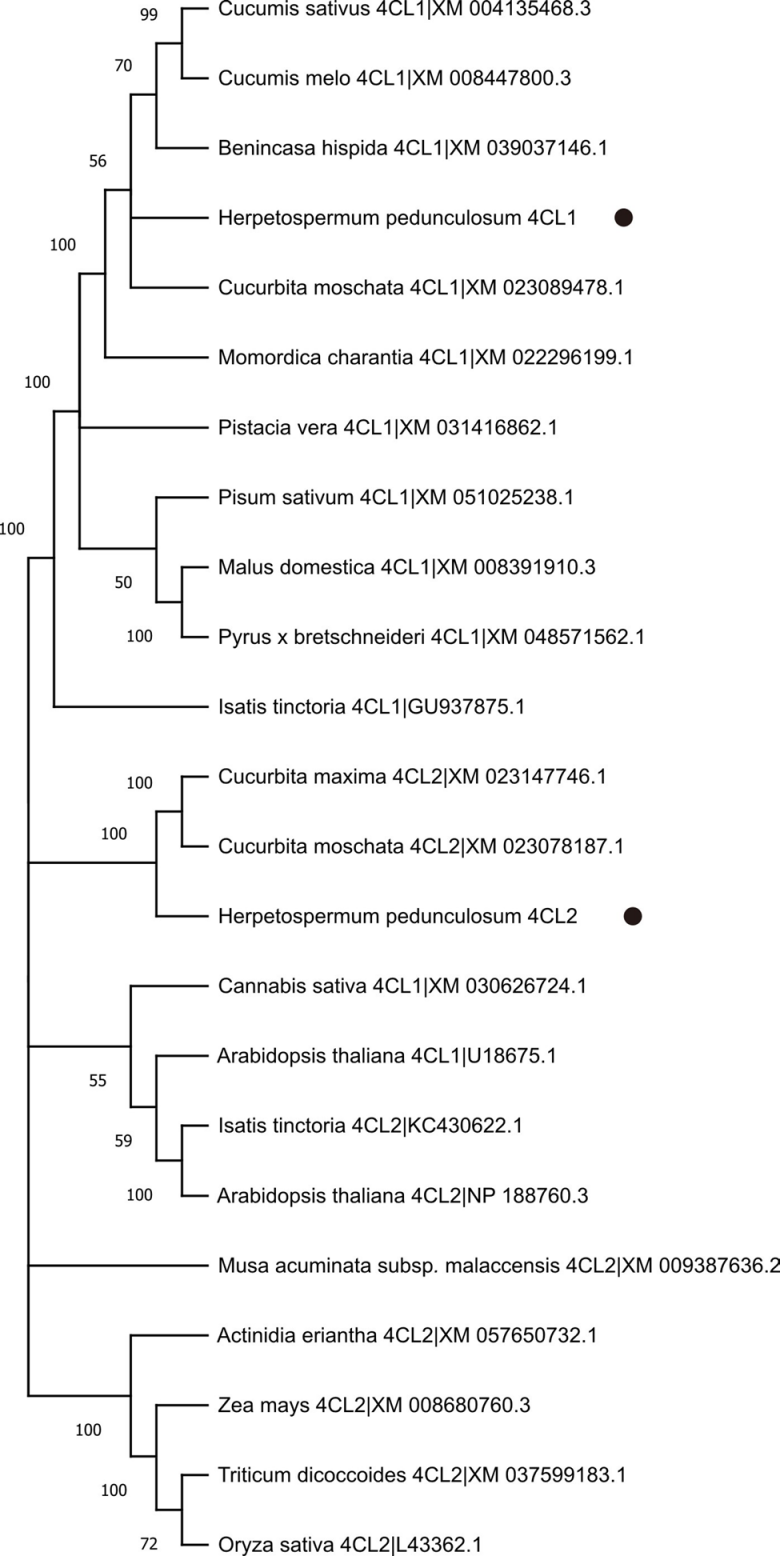
Phylogenetic relationships between Hp4CLs and 4CLs from different plants

**Fig. 10:**
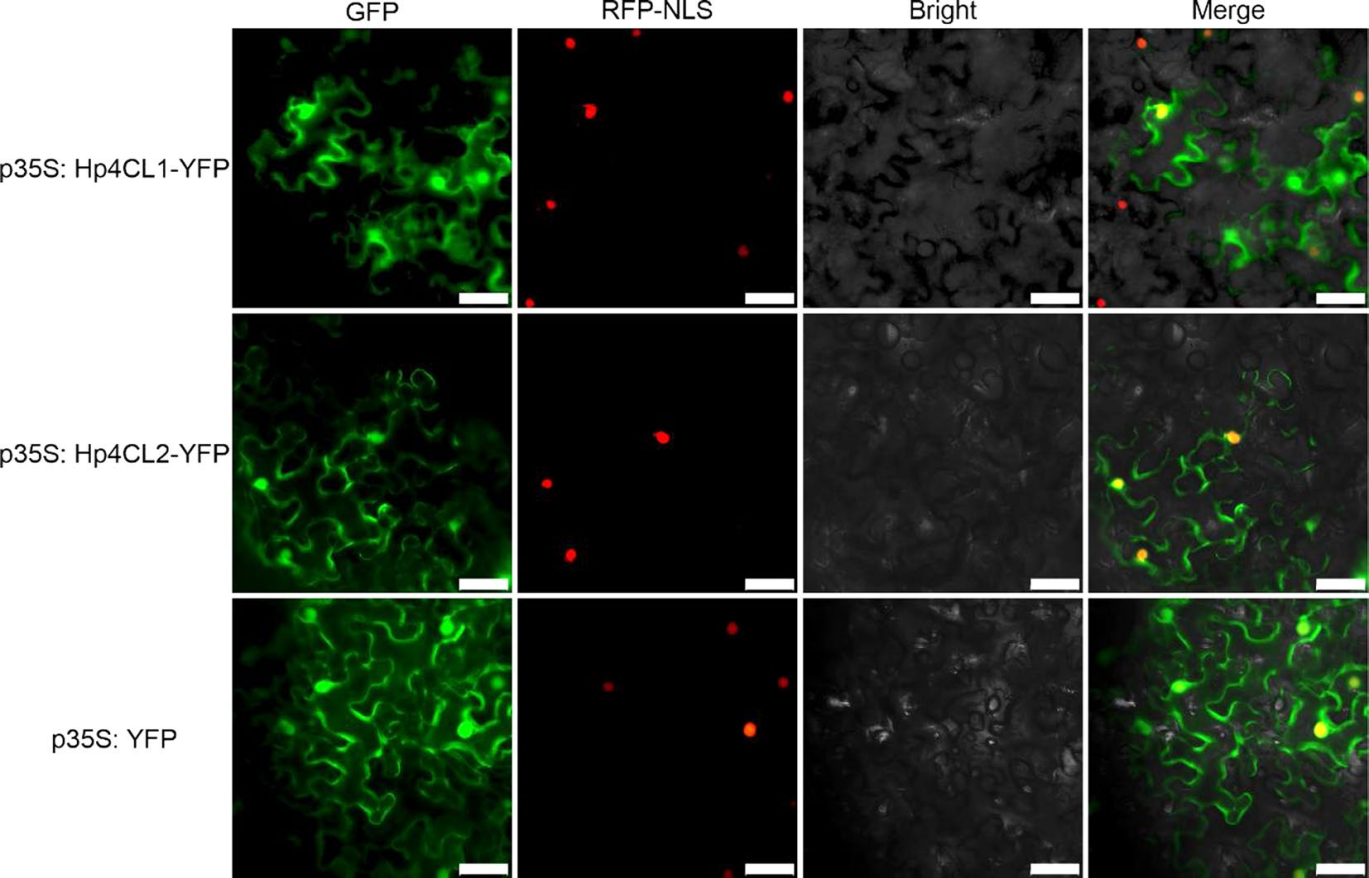
Subcellular localization of Hp4CL1 and Hp4CL2 in *N. benthamiana* leaves. Transiently expressing p35S: Hp4CL1-YFP or p35S: Hp4CL2-YFP fusion proteins in the leaves of *N. benthamiana* using *Agrobacterium tumefaciens*. Transient expression of p35S: YFP was used as a control. The subcellular localization of the fused proteins was analyzed using fluorescence microscopy 48 h after infiltration. RFP-NLS_SV40_ fusion protein serves as a nuclear marker. Scale bars, 50 μm

The ORFs of *Hp4CL1* and *Hp4CL2* were identified by PCR based on transcriptome sequences (Table S3). Hp4CL1 and Hp4CL2 shared high sequence homology with previously identified 4CL proteins. The amino acid residues essential for binding of AMP to the catalytic core were conserved in Hp4CL1 and Hp4CL2(Fig. 8) [26]. Phylogenetic analysis based on the deduced amino acid sequences of the two *Hp4CL* cDNAs showed that Hp4CL1 and Hp4CL2 belonged to class II, and their transcripts were expressed more in YM than in GM (Figs. 4D and 9). Class II 4CL are involved in the biosynthesis of phenolic compounds other than lignin [27]. Using the WoLF PSORT program, Hp4CL1 and Hp4CL2 were predicted to be localized to the cytosol and nucleus. To validate this result, yellow fluorescent protein (YFP)-fused Hp4CL1 or Hp4CL2 constructs were transiently expressed in *N. benthamiana* leaves to detect YFP signals. RFP-NLS_SV40_, a nuclear localization marker, was co-transformed with the Hp4CL fusion proteins. YFP observations showed that the fluorescence of Hp4CL1-YFP and Hp4CL2-YFP partially overlapped with that of RFP-NLS_SV40_, and Hp4CL1 and Hp4CL2 were localized in the cytosol and nucleus of *N. benthamiana* leaves, which was consistent with WoLF PSORT predictions (Fig. 10).

To ascertain the CoA ligase functions of Hp4CL1 and Hp4CL2, ORFs were cloned and expressed in *E. coli* BL21 (DE3). An enzymatic reaction test was performed on the purified recombinant Hp4CL1 and Hp4CL2 proteins (Fig. 10). 4CLs convert hydroxycinnamic acids to CoA esters but with varied substrate preferences [28]. Hp4CL1 showed a wide range of catalytic activities for all tested substrates. Hp4CL2 catalyzed *p*-coumaric acid, caffeic acid, and sinapic acid to produce the corresponding CoA esters. Enzyme kinetic analysis (Table 2) indicated that the highest affinities for Hp4CL1 and Hp4CL2 were for ferulic acid and sinapic acid, respectively. Hp4CL1 exhibited the highest catalytic efficiency for sinapic acid, whereas Hp4CL2 showed the highest catalytic efficiency for caffeic acid. As mentioned above, enzyme activity exhibited distinct catalytic characteristics for each 4CL.

**Fig. 11:**
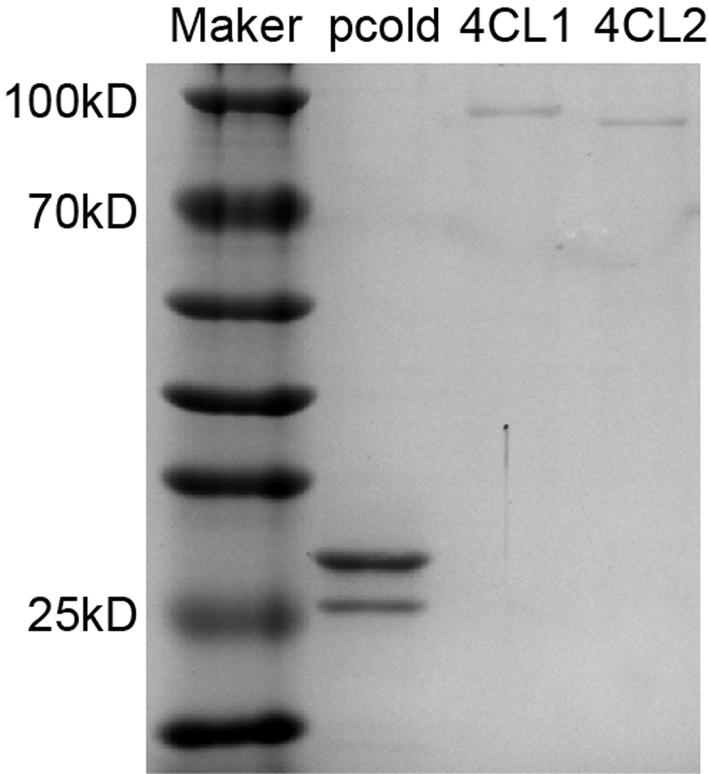
Purification of the two recombinant Hp4CL proteins. Proteins gel electrophoresis showed pCold-ProS2 empty vector and each purified 4CL protein with a calculated molecular weight of 23.1 kD, 86.7 kD, and 86.1 kD, respectively

**Table 2:** Kinetic analysis of Hp4CL1 and Hp4CL2

Protein name	Kinetic parameter	*p*-coumaric acid	Caffeic acid	Ferulic acid	Sinapic acid
	Kcat(s^− 1^)	0.447	0.242	1.034	0.197
	Km(mM)	1.561	0.720	3.272	0.456
Hp4CL1	Kcat/Km (s^− 1^ ·mM)	0.286	0.336	0.316	0.432
	Vmax (nkat mg^− 1^ Protein)	8.942	4.836	20.68	3.934
	Kcat(s^− 1^)	0.379	0.316	ND	0.477
Hp4CL2	Km(mM)	1.461	0.234	ND	1.838
	Kcat/Km (s^− 1^ ·mM)	0.259	1.352	ND	0.260
	Vmax (nkat mg^− 1^ Protein)	7.570	6.318	ND	9.540

ND: no detectable activity

## Discussion

The seeds of *H. pedunculosum* are rich in lignans and have incomparable medicinal value. The complete biosynthetic pathway for lignan production in *H. pedunculosum* remains obscure. Analyzing the chemical composition and gene expression of various seed development phases of medicinal plants using omics techniques can offer a valuable foundation for understanding the biosynthesis of lignan in these plants [16, 29]. Therefore, we performed a combined differential transcriptome and metabolome analysis of green and yellow mature-period seeds to understand the molecular mechanisms of lignan biosynthesis.

During the two stages of seed development, there was a significant alteration in the accumulation of metabolites associated with the phenylpropanoid (*p*-coumaric acid and coniferaldehyde) and lignan (syringin and secoisolariciresinol) pathways. *p*-coumaric acid is the main building block of phenylpropanoid metabolism, an intermediate product of lignan biosynthesis [30], and it is essential for the regulation of secondary metabolites such as lignans, lignins, and flavonoids [31]. A previous study has shown that flaxseed lignans are clustered with *p*-coumaric acid [32]. Therefore, *p*-coumaric acid may be regarded as an immediate precursor for lignan production. In this study, higher levels of *p*-coumaric acid were detected in YM seeds and the elevated expression of *4CL* may be attributed to an enhanced demand for *p*-coumaric acid for lignan biosynthesis (Table 1; Fig. 4D, Dataset S4). Previous researches have suggested that the most important hepatoprotective compounds in *H. pedunculosum* are lignans, primarily dehydrodiconiferyl alcohol, herpetrione, herpetin, herpetetrone and herpetotriol [9, 33]. Most of these species-specific lignans were found in YM seeds but not in GM seeds (Fig. 3). In addition, among the metabolites of YM and GM, seven identified lignan end-products were upregulated in YM compared with GM (Table 1). These results indicated that lignan accumulation mainly occurs during the late stages of seed development.

In parallel transcriptomic analysis, several key genes involved in the general phenylpropanoid pathway (e.g. *4CL*s, *HCT*, and *CCR2*) and lignan pathway (e.g. *POD*, *SDH*, and *DIR*) revealed similar upregulated patterns in correspondence with the above increased lignan-related metabolites (Fig. 5, Dataset S3), indicating that the transcriptomic and metabolomic profiles of the lignan biosynthesis pathway were altered. In higher plants, the biosynthesis of lignans is not only controlled by genes that are connected to catalytic processes but also involves a multitude of transcription factors. Several transcription factors, such as bHLH44, NAC54, MYB18, TNY, and EBP are involved in the regulation of lignan accumulation in *Isatis indigotica* [26, 34]. According to the TF-lignan-related metabolite network analysis, HSFB3 was highly correlated with all selected phenylpropanoid and lignan pathway DEMs (five upregulated and two downregulated), except for secoisolariciresinol (Fig. 7). Ten members of the heat shock factor (HSF) family had high correlations with phenylpropanoid content in *Narcissus tazetta* flowers [35]. In *Petunia hybrida*, HSF19 is associated with phenylpropanoid metabolism by positively regulating *PAL2* [36]. These results suggested that heat shock factor TFs may contribute to the transcriptional regulation of genes related to lignan synthesis in *H. pedunculosum* seeds. Additional research on the regulatory mechanisms of HSFB3 may offer valuable insights into the underlying mechanisms involved in lignan biosynthesis in *H. pedunculosum*.

To explore the mechanisms of *H. pedunculosum* lignan formation, a gene-metabolite pathway diagram was constructed (Fig. 6). Coniferyl alcohol is a universal precursor for all lignans [37]. This study demonstrated that a considerable number of genes involved in the phenylpropanoid pathway leading to coniferyl alcohols, such as *4CL1*, *4CL2*, *HCT*, and *CCR2*, were upregulated in YM vs. GM (Fig. 6, Dataset S4). Several studies have shown that genes related to the phenylpropanoid pathway affect the lignin accumulation [38–40]. Among them, *4CL1* and *4CL2* are *4CL* gene family members that play important roles in regulating the transfer of carbon from the phenylpropanoid metabolic pathway to the lignan, lignin, and flavonoid biosynthesis pathways [41]. In this study, the increased expression of *Hp4CL1* and *Hp4CL2* indicated a positive role for the phenylpropanoid metabolism pathway in the accumulation of lignans in YM. Among the two *4CL* gene family unigenes analyzed *Hp4CL1* and *Hp4CL2* encoded functional 4CL enzymes in *E. coli* strains expressing 4CL recombinant proteins (Fig. 11). Hp4CL1 was enzymatically characterized and demonstrated broad substrate specificity, catalyzing the formation of hydroxycinnamate-CoA thioesters from *p*-coumaric, ferulic, caffeic, and sinapic acids (Table 2). Hp4CL2 showed no catalytic activity toward ferulic acid (Table 2). Furthermore, the Hp4CL1 and Hp4CL2 proteins exhibited different catalytic efficiencies for all the tested substrates. Thus, Hp4CL1 and Hp4CL2 may have specific biochemical functions in the lignan biosynthesis.

Moreover, activation of the phenylpropanoid pathway is also linked to the upregulation of *DIR* and *POD*, which controls the initiation of species-specific lignan biosynthesis [16, 42, 43]. Heterologous expression of *PlDIR* from *Phryma leptostachya* stereoselectively couples coniferyl alcohol to form pinoresinol [23]. We found a DEG encoding a similar protein in *H. pedunculosum* (XP_023512165.1, E-value: 2 × 10^− 111^, percentage identity: 84.49%), which is a candidate for the stereo-configuration of a lignan. This activity remains to be demonstrated experimentally. Interestingly, this gene was upregulated in YM seeds compared with that in GM seeds. Therefore, the upregulation of genes related to the phenylpropanoid biosynthetic pathway at the seed maturation stage appeared to be responsible for the increased intermediate metabolite production and accumulation of species-specific lignans in *H. pedunculosum*.

Another aspect of lignan biosynthesis in *H. pedunculosum* requires uncovering the key genes involved in downstream steps from coniferyl alcohol to *Herpetospermum*-specific lignan biosynthesis. For this purpose, we generated a coniferyl alcohol backbone as the starting material to model this specific metabolic pathway, based on the basic principles of chemistry and biosynthesis (Fig. 6). We showed that herpetrione is the major lignan that accumulates in *H. pedunculosum*. For the formation of herpetrione, the phenyl-allyl alcohol portion of coniferyl alcohol undergoes two molecular addition and stereoselective dehydrogenation processes, resulting in the formation of (+)-pinoresinol. This process involves olefin addition and dehydrogenation. Based on the previous studies [44, 45], we speculated that HpDIR20 plays a key role in stereoselectivity, while related enzymes of the POD family may catalyze addition and dehydrogenation [46]. Next, the formed (+)-pinoresinol undergoes a similar addition and dehydrogenation process with another molecule of coniferyl alcohol (the reaction position is between the phenyl allyl alcohol of coniferyl alcohol and phenol parts of (+)-pinoresinol) to form an intermediate. Because these processes are similar, the enzymes involved are likely to belong to the POD family. The intermediate above contains a furan fragment, which is hydrolyzed and then dehydrogenated to obtain the final herpetrione. CYP is an enzymatic catalyst utilized in the structural modification of lignan end-products, leading to the generation of a diverse array of natural compounds [47]. Because CYPs are capable of catalyzing hydrolysis and dehydrogenation, we speculated that this process may be catalyzed by CYPs. For Herpetotriol, coniferyl alcohol first undergoes two-molecule addition and dehydrogenation processes under the promotion of POD family enzymes to form dehydrodiconifery alcohol. Dehydrodiconifery alcohol undergoes the same addition oxidation process as coniferyl alcohol, followed by demethoxylation promoted by ODM to obtain the final product. For Herpetin, it is possible that dehydrodiconiferyl alcohol and coniferyl alcohol undergo addition and oxidation reactions. Subsequently, they undergo a series of rearrangement and oxidation transformation processes, such as methyl migration, demethoxylation, addition and oxidation, and methoxylation, ultimately leading to the formation of herpetin. This information significantly contributes to a better understanding of *Herpetospermum* lignan biosynthesis and may expedite the implementation of biotechnological methods for lignan biosynthesis.

## Conclusions

**Fig. 12:**
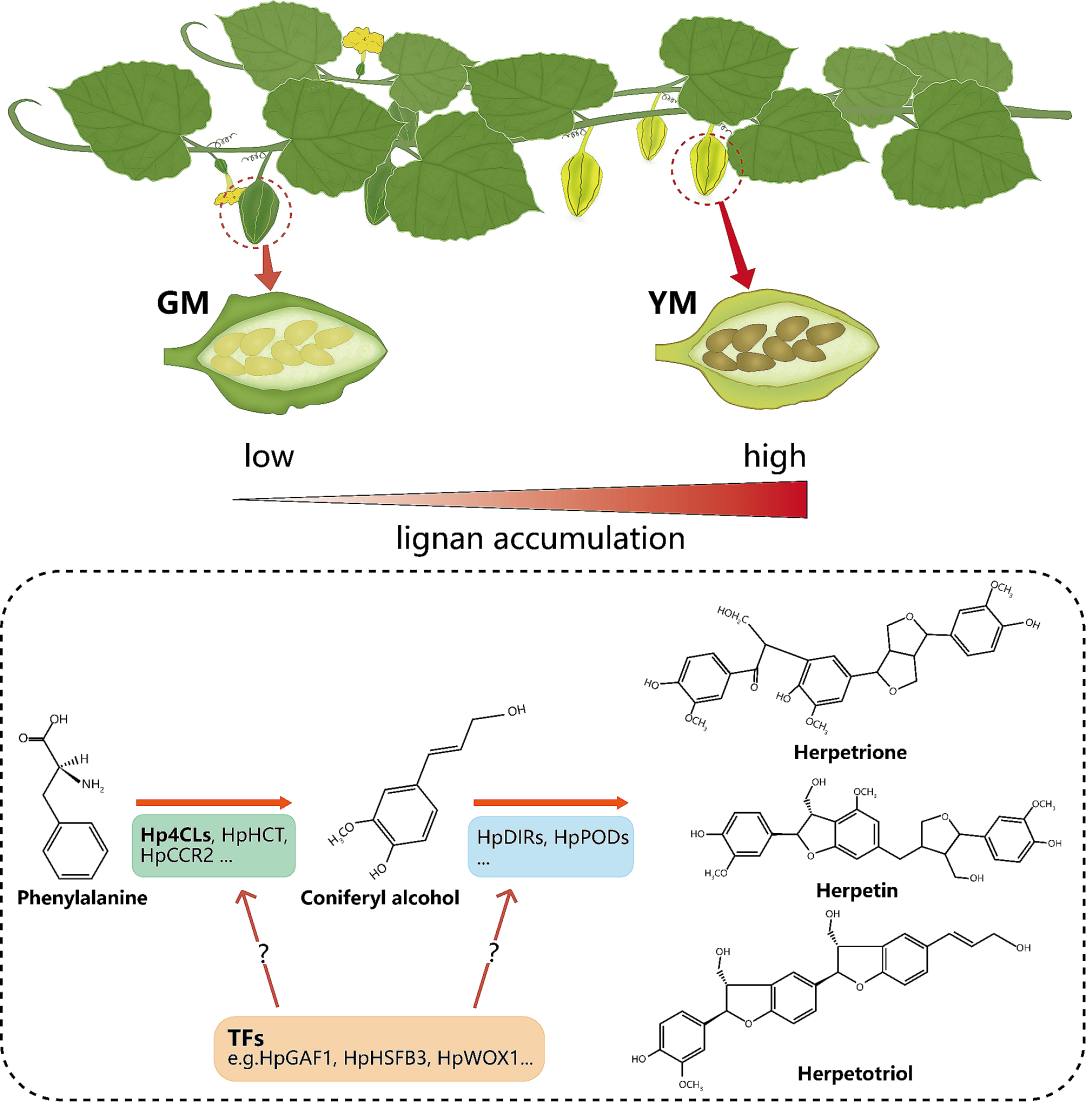
Schematic model of the mechanisms underlying high lignan accumulation in *H. pedunculosum* seeds. This model involves differentially expressed structural genes and TFs as key factors that positively regulate lignan accumulation

In conclusion, we performed an integrated metabolome and transcriptome analysis of the green and yellow mature stages of *H. pedunculosum*. A graphical summary of the mechanisms underlying high lignan accumulation in *H. pedunculosum* seeds is shown in Fig. 12. Because of the higher expression levels of genes involved in the phenylpropanoid and lignan biosynthesis pathways in YM seeds, there were significantly higher concentrations of lignans (especially Herpetrione, Eleutheroside E, Schisantherin E, and secoisolariciresino) in YM seeds than in GM seeds. Using correlation analysis, DEGs that showed expression levels strongly correlated with the concentrations of lignans were identified, including structural genes (e.g. *4CLs*, *HCT*, *CCR2*, *DIR*, and *POD*) and transcription factors, such as *GAF1*, *HSFB3*, and *WOX1*, might be involved in lignan biosynthesis. We also characterized the function of one of the most highly expressed rate-limiting synthases as 4-Coumarate: CoA ligase (4CL), using in vitro studies. Our work will provide new insights into the biosynthesis and accumulation of lignan in the Cucurbitaceae plant and also construct a correlation network of transcriptional expression levels and metabolite concentrations that could be used to apply genetic approaches to clarify the regulation mechanism of lignan.

## Materials and methods

### Plant materials

*H. pedunculosum* plants were artificially cultivated and collected from the cultivation site of the Tibet Rhodiola Pharmaceutical Holding Company, Lhasa, China. Seeds were harvested 30 and 60 days after pollination (Fig. 1), which corresponded to the GM and YM stage, respectively. The voucher specimens of GM (No. H-230618-1) and YM (No. No. H-230618-2) were authenticated by Dr. Qi Zhao (College of Food and Biological Engineering, Chengdu University) and deposited in the Engineering Research Center of Sichuan-Tibet Traditional Medicinal Plant, Chengdu, China. Freshly collected seeds were immediately stored in liquid nitrogen and stored at -80℃ until RNA extraction, metabolite extraction, and gene expression evaluation.

### Untargeted metabolic profiling analysis

The metabolite extraction and analysis were performed by Novogene Co. Ltd. (Beijing, China) following standard procedures described previously [48]. Partial least squares discriminant analysis (PLS-DA) was performed using metaX to identify the accumulation pattern of metabolites from 12seed samples (two time points and six biological conditions) [49]. Metabolites with variable importance in the project (VIP) > 1, *P*-value < 0.05, and fold change (FC) ≥ 2 or FC ≤ 0.5 were identified as differentially accumulated metabolites (DAMs). Volcano plots were used to filter metabolites of interest based on log_2_ (FC) and -log10 (*P*-value) of metabolites using ggplot2 in the R language. Metabolites were mapped to KEGG metabolic pathways for annotation and enrichment analysis [50].

### Lignan profile analysis

Lignan metabolite extraction and analysis were performed as previously described [9]. LC-MS was performed using a UPLC-Q-TOF/MS system (Shimadzu, Kyoto, Japan) to analyze the extracts from *H. pedunculosum* seeds and assess the *Herpetospermum*-specific lignan profile. The UPLC-ESI-QTOF/MS conditions were as follows: column, Shim-pack Scepter C18-120 column; electron impact mode, 70 eV; injection temperature, 250℃; column temperature, 160℃ at t = 0 min, and then increased to 250℃ at 20℃/min. The main lignans were identified using mixed standards (herpetrione, herpetotriol, and herpetin) as previously described [3]. The filtered fraction was analyzed by UPLC-Q-TOF/MS, and eluted with 0.1% aqueous formic acid (A) and acetonitrile (B) (0–5 min 28% B, 5–15 min 28–30% B, 15–20 min 30–40% B, 20–40 min 40–45% B, 40–41 min 45–95% B, 41–43 min 95% B, 43–45 min 28% B) at a flow rate of 1 ml/min.

### RNA-Seq profiling analysis

Total RNA was extracted at two stages (30 and 60 DAP) during the development of *H. pedunculosum* seeds using a Fast Plant RNA Extraction Kit (SENO, Zhangjiakou, China). RNA quality was evaluated using a Nanodrop microspectrophotometer (Thermo Scientific, Waltham, DE, USA) and Agilent 2100 bioanalyzer (Agilent Technologies, Santa Clara, USA). An RNA-Seq library with a 150 bp PE mode was then constructed and sequenced on the Illumina NovaSeq 6000 sequencing platform (Novogene, Beijing, China). The clean reads were mapped to the “*Herpetospermum pedunculosum*” reference genome [51]. The DEseq2 R package (version 1.20.0) was used to identify differentially expressed genes (DEGs) (|Log_2_ (FC)| > 1, padj < 0.05) between YM and GM. KEGG pathway enrichment studies were conducted using DEGs to examine the metabolic pathways and associated gene functions. The accumulation patterns of DEGs in YM vs. GM were analyzed using hierarchical cluster analysis (HCA).

### Gene expression analysis

To further verify the credibility and accuracy of the transcriptome data, we screened 12 DEGs associated with phenylpropanoid and lignan biosynthesis for expression levels in YM and GM using quantitative reverse transcription polymerase chain reaction (qRT-PCR). A Fast Plant RNA Extraction Kit (SENO, Zhangjiakou, China) and HiFiScript gDNA Removal RT MasterMix (Cwbio, Jiangsu, China) were used for RNA extraction and cDNA synthesis, respectively. qRT-PCR was carried out with the Cwbio SYBR Master Mix kit (Cwbio, Jiangsu, China) using the Bio-Rad CFX96 Real-time PCR system. Specific quantitative primers were obtained using the NCBI Primer-BLAST tool (https://www.ncbi.nlm.nih.gov/tools/primer-blast/) (Table S1). Relative expression levels were calculated using the 2^-ΔΔCT^ method, with actin as the internal standard. Three biological replicates and three technical replicates were used for all the qRT-PCR analyses.

### Integrated transcriptome and metabolome analysis

DAMs and DEGs were mapped onto the KEGG pathway map to better understand the relationships between the metabolites and genes. The metabolome and transcriptome data were integrated using Pearson correlation coefficients. Correlation coefficients with R^2^ > 0.9 (*P*-value < 0.05) were selected. Different lignan metabolites and DEG-encoding transcription factors (TFs) were selected for integrative analysis of lignan metabolism. Cytoscape (version 3.6.1) was used to visualize the final interaction network.

### Gene cloning, phylogenetic tree, and subcellular localization analysis

The open reading frame (ORF) of *Hp4CL1* and *Hp4CL2* were cloned from the cDNA of *H. pedunculosum* seeds using gene-specific primers (Table S1). The predicted polypeptide sequences were aligned using DNAMAN v.7.0.2. Neighbor-joining phylogenetic trees for 4CL proteins from *H. pedunculosum* and other plant species were constructed using MEGA 11 software with1000 bootstrap replicates and default settings (substitution type: amino acid; Model/Method: Jones-Taylor-Thornton (JTT) model; rates among sites: Uniform Rates; Gaps/Missing Data Treatment: Complete deletion). The protein subcellular localization of 4CLs was predicted using WoLF PSORT (https://www.genscript.com/wolf-psort.html, accessed on 8 August 2023). For subcellular localization analysis, the ORFs of *Hp4CL1* and *Hp4CL2* without termination codons were subcloned into the pCAMBIA1300-35 S-YFP vector. All the primers used are listed in Table S1. The marker protein RFP-NLS_SV40_ was used to locate the proteins in the nucleus [52]. Instantaneous transformations and fluorescence signals were observed, as previously described [53].

### Prokaryotic expression and enzyme activity assays

The ORFs of *Hp4CL1* and *Hp4CL2* were subcloned into the pCold-ProS2 expression vector. All primers used are listed in Table S1. The constructed empty vectors pCold-ProS2, pCold-ProS2-Hp4CL1, and pCold-ProS2-Hp4CL2 were transformed into *E. coli* BL21(DE3)-competent cells for recombinant protein expression. Cell cultivation and collection were performed as previously described [54]. Cells were harvested by centrifugation at 12,000 rpm and resuspended in 200 mM phosphate-buffered saline (pH 7.5) before ultrasonication. The supernatant containing Hp4CL1 or Hp4CL2 was subjected to a protein purification assay using the BeaverBeads His-tag Protein Purification Kit (BeaverBeads, China) following the manufacturer’s instructions. The purity of the His-tag recombinant proteins was examined using 12% (w/v) SDS-PAGE, and the concentration was determined using the Bradford method.

The enzyme activity was measured following a previously described method [26]. A 500 µM solution of *p*-coumaric acid, caffeic acid, ferulic acid, and sinapic acid was used as the donor substrate to measure the catalytic activities of Hp4CL1 and Hp4CL2. In addition, 100 mM Tris–HCl (pH 7.5), 2.5 mM ATP, 2.5 mM MgCl_2_, 0.2 mM CoA, and 0.2 mM CoA were also included in the reaction system with 10 µg/ml purified protein. The production of CoA esters was measured by UV spectrophotometry. Changes in absorbance were monitored at wavelengths of 311, 333, 345, 346, and 352 nm for the production of *p*-coumaroyl-CoA, feruloyl-CoA, caffeoyl-CoA, and sinapoyl-CoA, respectively. Vmax and Km values were determined from Lineweaver–Burk plots, and kcat was determined by dividing Vmax by the enzyme concentration. Each reaction was performed in triplicates.

### Electronic supplementary material

Below is the link to the electronic supplementary material.

Supplementary Material 1

Supplementary Material 2

Supplementary Material 3

## Data Availability

The raw RNA-seq read data are deposited in the BioProject, PRJNA1061772(https://www.ncbi.nlm.nih.gov/bioproject/PRJNA1061772). Metabolome data are available on FigShare at the link: https://doi.org/10.6084/m9.figshare.24906093.
