# Supplementary material for: Integrative analysis of the metabolome and transcriptome provides insights into the mechanisms of lignan biosynthesis in Herpetospermum pedunculosum (Cucurbitaceae)
Source: BMC Genomics. 2024 Apr 29;25:421. doi: 10.1186/s12864-024-10306-1 (PMC11059704; doi:10.1186/s12864-024-10306-1)
Supplement: Supplementary file 2 — Supplementary Material 2 [file 12864_2024_10306_MOESM2_ESM.docx]

Table S1. Primers used in this study.

| Primer name | Sequence (5’-3’) | Usages |
| --- | --- | --- |
| Hp4CL1-qRT-F | GGAATTTGATCTTAGCGGTCTC | qRT-PCR |
| Hp4CL1-qRT-R | CCATATGCCTCTTGAACATCC | qRT-PCR |
| Hp4CL2-qRT-F | GGGTGAAGATTTTGTGTACGG | qRT-PCR |
| Hp4CL2-qRT-R | GTAGTTCCAGACGAAAACGG | qRT-PCR |
| Hp4CL6-qRT-F | GCACGGTTCCTAAATGATCAC | qRT-PCR |
| Hp4CL6-qRT-R | ACGACTACGACAGATTCCTG | qRT-PCR |
| HpCOMT1-qRT-F | CGCCCGTTTCTAAGTATTTTG | qRT-PCR |
| HpCOMT1-qRT-R | CATGAGCCCTGTTGAAAGG | qRT-PCR |
| HpCCoAOMT-qRT-F | CGATCTGGTCAAAGTTGGTG | qRT-PCR |
| HpCCoAOMT-qRT-R | CACGAAGTCCCTGTAATACC | qRT-PCR |
| HpCCR1-qRT-F | CTGGTTACTCTGACTATGTCG | qRT-PCR |
| HpCCR1-qRT-R | CCTCCATCTTTTGATCTTGCC | qRT-PCR |
| HpCCR2-qRT-F | GCAGTCATCGGAACAAAGAAT | qRT-PCR |
| HpCCR2-qRT-R | CACTCCAACATGTCTCATCC | qRT-PCR |
| HpCAD1-qRT-F | TCCCTGTTTCTCATCCTCTC | qRT-PCR |
| HpCAD1-qRT-R | CCCTGTGATCGTCTTCCTC | qRT-PCR |
| HpCAD9-qRT-F | TGTTTCTCTAGTTCTCGGTAGG | qRT-PCR |
| HpCAD9-qRT-R | GCCGTGTTAATCTCGTTCATC | qRT-PCR |
| HpDIR19-qRT-F | ACAACTTCCCCATCCTCG | qRT-PCR |
| HpDIR19-qRT-R | GCTTTTCCCCACAGTTTTG | qRT-PCR |
| HpDIR20-qRT-F | CGAGATTGGGCTTTTGATGG | qRT-PCR |
| HpDIR20-qRT-R | AGGATGGTGATCGAGCTG | qRT-PCR |
| HpSDH-qRT-F | GCAAAACTCTTCGTCCACC | qRT-PCR |
| HpSDH-qRT-R | GCGACTTGGATTTGGGATTC | qRT-PCR |
| HpPOD2-qRT-F | GCCTTGATTCCACTGATCTTG | qRT-PCR |
| HpPOD2-qRT-R | TGGGTCTAGTGTTGGATCTG | qRT-PCR |
| HpActin-qRT-F | GGGTTCATAAAGGATTGGGATG | qRT-PCR |
| HpActin-qRT-R | TGGGAGTAGAAAGTGGATCTG | qRT-PCR |
| Hp4CL1-p1300-YFP-F | acgggggacgagctcggatccATGGCTACTTGCATTCGAGATTC | Subcellular location |
| Hp4CL1-p1300-YFP-R | gcccttgctcaccatggtaccAGAGGCAGCCTCGGCTCGAA | Subcellular location |
| Hp4CL2-p1300-YFP-F | acgggggacgagctcggatccATGGCGACACCCGATCCGT | Subcellular location |
| Hp4CL2-p1300-YFP-R | gcccttgctcaccatggtaccATTTGGAATACTGGCTGCGAG | Subcellular location |
| Hp4CL1-pCold ProS2-F | gaaggtaggcatatggagctcATGGCTACTTGCATTCGAGATTC | Recombined protein |
| Hp4CL1-pCold ProS2-R | gacaagcttgaattcggatccTCAAGAGGCAGCCTCGGC | Recombined protein |
| Hp4CL2-pCold ProS2-F | gaaggtaggcatatggagctcATGGCAACGCCCGATCCG | Recombined protein |
| Hp4CL2-pCold ProS2-R | gacaagcttgaattcggatccCTAATTTGGAATACTGGCTGCGA | Recombined protein |

Table S2. Summary of the assembly of transcriptome

| **sample** | **library** | **raw_reads** | **raw_bases** | **clean_bases** | **error_rate** | **Q20** | **Q30** | **GC_pct** |
| --- | --- | --- | --- | --- | --- | --- | --- | --- |
| **YM_1** | YM1 | 20997125 | 6.3G | 6.23G | 0.03 | 97.33 | 92.6 | 47.21 |
| **YM_2** | YM2 | 21010819 | 6.3G | 6.22G | 0.03 | 97.16 | 92.31 | 46.94 |
| **YM_3** | YM3 | 21267124 | 6.38G | 6.3G | 0.03 | 97.26 | 92.48 | 45.16 |
| **GM_1** | GM1 | 21398311 | 6.42G | 6.35G | 0.03 | 97.37 | 92.75 | 46.29 |
| **GM_2** | GM2 | 21147034 | 6.34G | 6.29G | 0.03 | 96.96 | 91.72 | 44.56 |
| **GM_3** | GM3 | 21439032 | 6.43G | 6.38G | 0.03 | 97.52 | 93.03 | 44.52 |

Table S3. Sequence of ORF used for functional validation of *HP4CL* genes.

| ORF for functional validation of *HP4CL1* |
| --- |
| ATGGCTACTTGCATTCGAGATTCGGTAGAAGACGAGGAACACATTTTCCGCAGCCAACTTCCTGAGGTCCAAGTGCCTGATGATATTACATTGCCGGAGTTTGTACTTCAGAATGCTGAATCATATGCTGAAAATGTGGCATTTGTGGAAGCCGTGAGCGGAAAGGCGTATACTTACCGCGAAGTTGTGAGAGATACGAATAGATTCGCTAAGGCCTTGAGTTCTCTGAGGTTGAGGAAGGGGCAGGTGGTTATTGTTGTTCTACCTAACGTTGCAGAATATGCCATTGTTGCTTTAGGCATAATGGCTGCTGGAGGTGTGTTTTCTGGTGTGAATCCAGCAGCTCACATATCAGAAATTCAAAAGCAGGTGGAGACAGCAGAAGCCAAACTCGTCGTCACAAACAGTGCAAACTTTGAAAAGGTGAAGGAATTAAAGCTACCAGTGATCGTATTGGGGGAGGAACTGATTGAAGGTTCCATGAACTGGCACAAACTGCTTGAAGCTGCGGACCGTGCAGGCAACAATTTTGTCAAAGAAGATATCAAGCAGACTGATTTATGTGCACTTCCTTTCTCATCAGGAACCACAGGGGTTTCCAAAGGCGTTATGCTAACTCACAGAAATATAGTGGCTAACTTGTGTTCTACTCTCTCCGGTGTGCCGCAAGAAATGGTCGGCAAGGTCACGACGTTAGGCCTCATTCCGTTCTTCCACATTTACGGGATTACTGGAATATGTTGTGCCACACTTAGAAACAAGGGAAAAGTTGTGGTGATGGGAAGATTTGATCTCAGGACTTTCATTAATGCCCTCATAACACAGGAGGTCACATTTGCTCCAATTGTTCCTCCTATCATCCTGGCTTTGGTTAAGAATCCTATTGTGGAGGAATTTGATCTTAGCGGTCTCAAACTTCAGGCTATCATGACTGCAGCTGCTCCGCTCGCACCGGAACTTCAAACTGCCTTTGAGAAAAAGTTCCCGGGCGTGGATGTTCAAGAGGCATATGGACTAACCGAGCACAGCTGCATCACTCTCAACTATGGAAGTATAGGAAAAGAGAATCTCGCTACAAAGAAAAACACAGTTGGCTGCATTCTTCCTAACCTAGAAGTCAAATTCATTGACCCCGACAGCGGCAGGTCTCTGCCAAAGAATACTCCGGGTGAAGTCTGTGTAAGAAGCCAGTGTGTGATGCAAGGCTACTACAAAAATGAAGAGGAGACTTCAAGGACCATTGACAACAAAGGCTGGATGCACACTGGCGACATCGGATACATTGATGACGATGGCGATGTGTTTATTGTGGATCGTATTAAAGAATTGATCAAGTACAAAGGCTTTCAAGTTGCCCCAGCTGAGTTAGAGGCAATCCTCCTGACTCATCCCTCCATTGAAGATGCAGCTGTGGTGCCGCTGCCAGACGAAGAAGCCGGTGAGATCCCAGCAGCAAGCGTCGTGATAGCTCTGAATTCAAATGAAACTGAAGATGAAATAATGAAATACGTTGCCTCAAACGTTGCACATTACAAGAAAGTTAGGCTGGTTCATTTTGTGGACACTATCCCAAAATCGCCTTCTGGGAAAGTTATGAGAAGGTTGATCAAAGAGAAGATGATCGAAAAGATTCGAGCCGAGGCCGCCTCTTGA |
| ORF for functional validation of *HP4CL2* |
| ATGGCGACGCCCGATCCGTCACCGGAATTCATATTCCGATCAAAACTGCCGGACATCTTCATCCCCAACCACCTCCCCTTGCACACATATTGCTTCGAAAAGATCTCCGAATTCAAACACCGTCCATGTCTAATCAACGCCGCCACCGGCGACACCCACACCTACGCCGAAGTCCACGCAACATCCCGGCGAGTCGCCGCCGGTCTCCACAAGCTCGGCATCGGAAAAGGCGACGTAATCATGCTCCTCCTCCAAAACAGCCCACAGTTCGTCCTAGCTTTCCTCGGCGCCTCCATTGTCGGTGCCGCCTCCACCACGGCGAACCCATTCTTCACACCACCGGAGATCGCAAAACAAGCAGCGTCCTCCGGCGCAAAGCTGATCATAACCCAAGCTGCATTCGCCGAGAAAGTGAAAAACCTCCGTCATGAAAACGGCGTGATCATCAAAGTCATATTCATCGACGACGACGGTGGGTCCCATTTTTCGGTTTTGACCGATCACGGAAAAGAAGAGGAAATAATAATCCCCGATGTGAAAATCAGTCCAAACGACGTCGTTGCGCTGCCTTATTCATCTGGGACCACAGGGTTACCAAAGGGAGTGATGCTGACTCATAAGGGATTGGTGACCAGCGTGGCGCAACAGGTGGACGGTGAGGATCCGCACCTCCACATCGGATGCAACGACGTCGTTTTGTGCGTGCTTCCTTTGTTTCACATATATTCGCTGAACTCGATCATGATGTGTTCGTTGCGAGTCGGAGCCGCGATTTTGATCGTACAGAAATTCGAAATGAATTCAATTGTGGAGTTGGTGCCGAAATATAAGGTGACGATAGCGCCGTTTGTGCCTCCGATTGTATTGGCGATTGCCAAGAGTCCGGCGGTGGAGGGTTCCGACATGTCGTCGATACGGATGGTGTTGTCGGGAGCGGCACCGCTGGGAAAGGATCTTGAAGATGCCTTTAGAGCCAAACTTCCACATGCCATACTCGGACAGGGATATGGAATGACGGAATCGGGGTCGGTGATGACGATGTCGTTGGCGTTTGCAAAAGAGAAGTTCGAAGTAAAATCGGGAGGTTGTGGGACATTGATGAGAAATTCAGAGATGAAGATCATAAATCCTCAAACGGGAGCTTCTCTTCCAAGGAATCAACCTGGGGAGATTTATGTTAGAAGTCCTCAGTTGATGAAAGGATACCTCAACGACGAAGCGGCCACGAAGGGCATAATCGACGAAGACGGATGGCTGCACACCGGCGACATAGGTTTCGTCGACGACGACGACGAGGTCTTCATCGTCGATCGCCTCAAGGAACTGATCAAATACAAAGGCTTCCAGGTGGCGCCGGCGGAGTTGGAAGCTCTCCTGATTTCCCACAGTCACATCGCTGACGCTGCGGTTATACCTATGAAAGATGAAGTTGCTGGAGAAGTTCCGGTTGCGTTCATTGTTCGATCCGACGGTTCCAACATCACCGAGGATGAAATTAAGCAATTCATCTCCAAACAGGTTGTGTTTTATAAGAGGATTAATAGTGTCTTCTTCGTGGATTCCATTCCTAAAAGCCCTTCTGGAAAAATCTTGCGGAGACAACTTAGAGCTTTGCTCGCAGCCAGTATTCCAAATTAG |
