# Supplementary material for: Integrative analysis of the metabolome and transcriptome provides insights into the mechanisms of lignan biosynthesis in Herpetospermum pedunculosum (Cucurbitaceae)
Source: BMC Genomics. 2024 Apr 29;25:421. doi: 10.1186/s12864-024-10306-1 (PMC11059704; doi:10.1186/s12864-024-10306-1)
Supplement: Supplementary file 3 — Supplementary Material 3 [file 12864_2024_10306_MOESM3_ESM.docx]

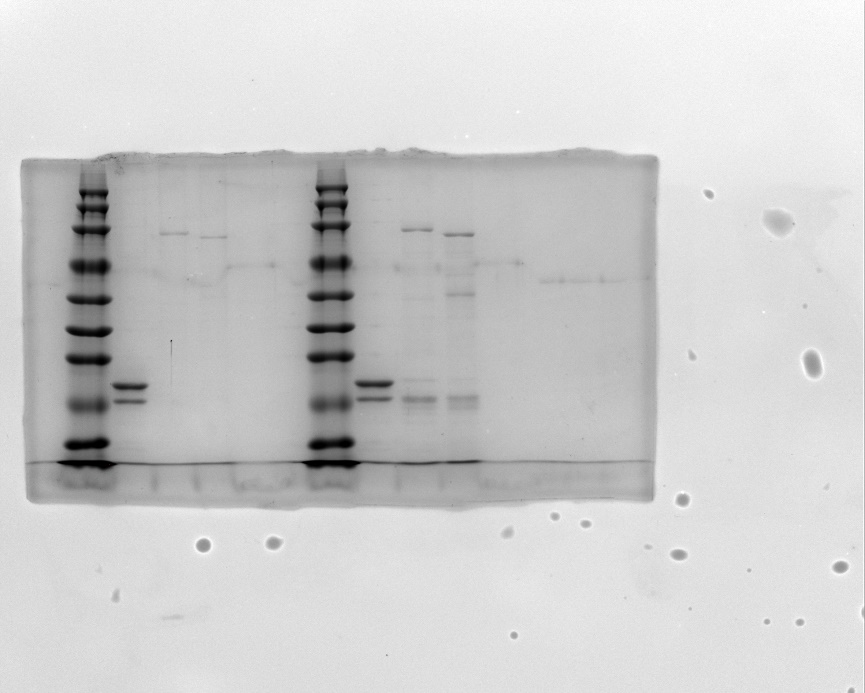


Figure S1. The full uncropped gel of purification of the two recombinant Hp4CL proteins. The lane 1-4 were the areas used in the Fig. 11.
